# (3*Z*)-3-Hydrazinylideneindolin-2-one

**DOI:** 10.1107/S1600536811035367

**Published:** 2011-09-14

**Authors:** Rifat Ara Jamal, Uzma Ashiq, Sammer Yousuf

**Affiliations:** a Department of Chemistry, University of Karachi, Karachi 75270, Pakistan; b H.E.J. Research Institute of Chemistry, International Center for Chemical and Biological Sciences, University of Karachi, Karachi 75270, Pakistan

## Abstract

The title mol­ecule, C_8_H_7_N_3_O, is almost planar, with a maximum deviation of 0.0232 (2) Å from the least-squares plane. The *Z* conformation of the C=N double bond is stabilized by an intra­molecular N—H⋯O hydrogen bond. In the crystal, adjacent mol­ecules are linked by inter­molecular N—H⋯N and N—H⋯O hydrogen bonds, forming zigzag sheets parallel to the *c* axis; the sheets are further stabilized by π–π inter­actions [centroid–centroid distance = 3.7390 (10) Å].

## Related literature

For the biological activity of related compounds, see: Sarangapani *et al.* (1994 ▶). For related structures, see: Ali *et al.* (2005*a* ▶,*b* ▶); Pelosi *et al.* (2005 ▶).
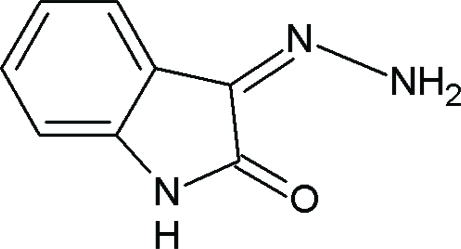


## Experimental

### 

#### Crystal data

C_8_H_7_N_3_O
*M* *_r_* = 161.17
Orthorhombic, 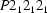

*a* = 4.7211 (5) Å
*b* = 11.4263 (13) Å
*c* = 13.3693 (15) Å
*V* = 721.20 (14) Å^3^
*Z* = 4
Mo *K*α radiation
μ = 0.10 mm^−1^
*T* = 273 K
0.50 × 0.10 × 0.09 mm

#### Data collection

Bruker SMART APEX CCD area-detector diffractometer
Absorption correction: multi-scan (*SADABS*; Bruker, 2000 ▶) *T*_min_ = 0.950, *T*_max_ = 0.991
4234 measured reflections
811 independent reflections
776 reflections with *I* > 2σ(*I*)
*R*_int_ = 0.021

#### Refinement

*R*[*F* ^2^ > 2σ(*F* ^2^)] = 0.030
*wR*(*F* ^2^) = 0.079
*S* = 1.08
811 reflections
121 parameters
H atoms treated by a mixture of independent and constrained refinement
Δρ_max_ = 0.12 e Å^−3^
Δρ_min_ = −0.16 e Å^−3^

### 

Data collection: *SMART* (Bruker, 2000 ▶); cell refinement: *SAINT* (Bruker, 2000 ▶); data reduction: *SAINT*; program(s) used to solve structure: *SHELXS97* (Sheldrick, 2008 ▶); program(s) used to refine structure: *SHELXL97* (Sheldrick, 2008 ▶); molecular graphics: *SHELXTL* (Sheldrick, 2008 ▶); software used to prepare material for publication: *SHELXTL*, *PARST* (Nardelli, 1995 ▶) and *PLATON* (Spek, 2009 ▶).

## Supplementary Material

Crystal structure: contains datablock(s) global, I. DOI: 10.1107/S1600536811035367/ng5222sup1.cif

Structure factors: contains datablock(s) I. DOI: 10.1107/S1600536811035367/ng5222Isup2.hkl

Supplementary material file. DOI: 10.1107/S1600536811035367/ng5222Isup3.cml

Additional supplementary materials:  crystallographic information; 3D view; checkCIF report

## Figures and Tables

**Table 1:** Hydrogen-bond geometry (Å, °)

*D*—H⋯*A*	*D*—H	H⋯*A*	*D*⋯*A*	*D*—H⋯*A*
N3—H2*N*3⋯O1	0.88 (2)	2.09 (2)	2.784 (2)	135 (2)
N3—H1*N*3⋯N2^i^	0.91 (2)	2.20 (3)	3.098 (2)	169 (2)
N1—H1*N*1⋯O1^ii^	0.90 (2)	1.98 (2)	2.866 (2)	168 (3)

Symmetry codes: (i) 
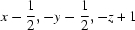
; (ii) 
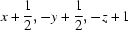
.

**Fig. 1.**
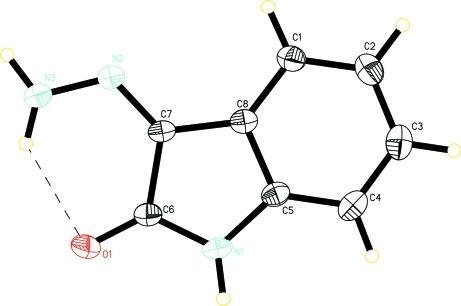
The molecular structure of (I) with displacement ellipsoids drawn at 50% probability level. The dashed lines indicates the intramolecular hydrogen bonds.

**Fig. 2.**
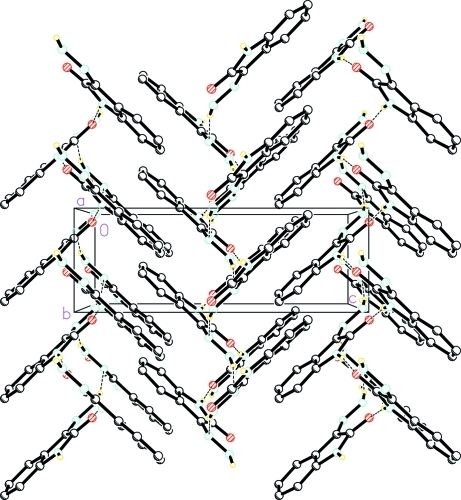
The crystal packing of the title compound I. Only hydrogen atoms involved in hydrogen bonding are shown.

## supplementary crystallographic information

### Comment

Isatins are very important compounds due to their antifungal properties (Sarangapani & Reddy, 1994). In view of this biological significance, the crystal structure of the title compound has been determined (Fig. 1). The title compound I was found to be antifungal and phytotoxic (U. Ashiq & R.A. Jamal, unpublished results).

The title structure consists of a hydrazine group and indole ring linked by C=N bond exist in *Z* conformation. The molecule is essentially planar with a maximum deviation of 0.0232 (2) Å from the least-square plane. The *Z* conformation of the olefinic bond is get stablized by N3—H2N3···O1 intramolecular hydrogen bond (Fig. 1). The bond lengths and angles all are in normal range as in other structurally related compounds (Ali *et al.*, 2005*a*,2005*b*; Pelosi *et al.*, 2005)]. In the crystal structure, the molecules are linked by N3—H1N3···N2 and N1—H1N1···O1 intermolecular hydrogen bonds to form zig zag sheets running parallel to *c* axis. (symmetry codes as in Table 1, Fig. 2). The intermolecular interactions network is further strengthened by significant π–π interactions between pyrrole (*Cg*(1)= N1/C5–C8) and phenyl (*Cg*(2)= C1–C5/C8) rings; (*Cg*(1)to *Cg*(2) distance = 3.7390 (10) Å; -1+*X*,Y,*Z*).

### Experimental

To a solution of 2,3-Indolinedione (25 mmol, 3.67 g) in 30 ml of ethanol with few drops of glacial acetic acid, hydrazine hydrate (12.5 ml, 250 mmol), was added. The mixture was refluxed for 2 h and a solid was obtained upon removal of the solvent by rotary evaporation. Crystal of the title compound suitable for X-ray crystallographic study were grown from a solution of ethanol by slow evaporation at room temperature.

### Refinement

H atoms on the C of methine were positioned geomatrically with C–H= 0.93 Å, and constrained to ride on their parent atoms with *U*_iso_(H)= 1.2*U*_eq_(CH). The H atoms on the N atoms (N–H= 0.91 (2)–0.886 (19) Å) atoms were located in difference Fourier maps and refined isotropically. During refinement 521 Friedel pairs were merged.

### Crystal data

**Table:**

C_8_H_7_N_3_O	*D*_x_ = 1.484 Mg m^−^^3^
*M**_r_* = 161.17	Mo *K*α radiation, λ = 0.71073 Å
Orthorhombic, *P*2_1_2_1_2_1_	Cell parameters from 2104 reflections
*a* = 4.7211 (5) Å	θ = 3.1–27.5°
*b* = 11.4263 (13) Å	µ = 0.10 mm^−^^1^
*c* = 13.3693 (15) Å	*T* = 273 K
*V* = 721.20 (14) Å^3^	Plate, colorles
*Z* = 4	0.50 × 0.10 × 0.09 mm
*F*(000) = 336	

### Data collection

**Table:**

Bruker SMART APEX CCD area-detector diffractometer	811 independent reflections
Radiation source: fine-focus sealed tube	776 reflections with *I* > 2σ(*I*)
graphite	*R*_int_ = 0.021
ω scans	θ_max_ = 25.5°, θ_min_ = 2.3°
Absorption correction: multi-scan (*SADABS*; Bruker, 2000)	*h* = −5→5
*T*_min_ = 0.950, *T*_max_ = 0.991	*k* = −13→13
4234 measured reflections	*l* = −16→15

### Refinement

**Table:**

Refinement on *F*^2^	Primary atom site location: structure-invariant direct methods
Least-squares matrix: full	Secondary atom site location: difference Fourier map
*R*[*F*^2^ > 2σ(*F*^2^)] = 0.030	Hydrogen site location: inferred from neighbouring sites
*wR*(*F*^2^) = 0.079	H atoms treated by a mixture of independent and constrained refinement
*S* = 1.08	*w* = 1/[σ^2^(*F*_o_^2^) + (0.0512*P*)^2^ + 0.0832*P*] where *P* = (*F*_o_^2^ + 2*F*_c_^2^)/3
811 reflections	(Δ/σ)_max_ < 0.001
121 parameters	Δρ_max_ = 0.12 e Å^−^^3^
0 restraints	Δρ_min_ = −0.16 e Å^−^^3^

### Special details

**Table:**

Geometry. All e.s.d.'s (except the e.s.d. in the dihedral angle between two l.s. planes) are estimated using the full covariance matrix. The cell e.s.d.'s are taken into account individually in the estimation of e.s.d.'s in distances, angles and torsion angles; correlations between e.s.d.'s in cell parameters are only used when they are defined by crystal symmetry. An approximate (isotropic) treatment of cell e.s.d.'s is used for estimating e.s.d.'s involving l.s. planes.
Refinement. Refinement of *F*^2^ against ALL reflections. The weighted *R*-factor *wR* and goodness of fit *S* are based on *F*^2^, conventional *R*-factors *R* are based on *F*, with *F* set to zero for negative *F*^2^. The threshold expression of *F*^2^ > σ(*F*^2^) is used only for calculating *R*-factors(gt) *etc*. and is not relevant to the choice of reflections for refinement. *R*-factors based on *F*^2^ are statistically about twice as large as those based on *F*, and *R*- factors based on ALL data will be even larger.

### Fractional atomic coordinates and isotropic or equivalent isotropic displacement parameters (Å^2^)

**Table:**

	*x*	*y*	*z*	*U*_iso_*/*U*_eq_	
O1	0.1159 (3)	0.11147 (11)	0.46693 (10)	0.0443 (4)	
N2	0.2067 (3)	−0.13322 (13)	0.54465 (10)	0.0345 (4)	
N3	0.0172 (4)	−0.12868 (16)	0.47156 (13)	0.0429 (4)	
C8	0.5392 (4)	−0.02667 (15)	0.65275 (13)	0.0331 (4)	
C6	0.2809 (4)	0.08289 (15)	0.53444 (14)	0.0343 (4)	
C7	0.3271 (4)	−0.03705 (14)	0.57432 (13)	0.0317 (4)	
N1	0.4577 (4)	0.15444 (13)	0.58597 (13)	0.0405 (4)	
C1	0.6635 (5)	−0.10479 (17)	0.71866 (14)	0.0408 (5)	
H1A	0.6159	−0.1837	0.7167	0.049*	
C4	0.8090 (5)	0.13277 (19)	0.72546 (15)	0.0460 (5)	
H4A	0.8570	0.2117	0.7277	0.055*	
C2	0.8591 (5)	−0.06419 (19)	0.78751 (15)	0.0469 (5)	
H2B	0.9425	−0.1160	0.8323	0.056*	
C5	0.6139 (4)	0.09186 (16)	0.65757 (14)	0.0357 (5)	
C3	0.9315 (5)	0.0535 (2)	0.79003 (16)	0.0491 (6)	
H3A	1.0651	0.0794	0.8361	0.059*	
H2N3	−0.028 (5)	−0.060 (2)	0.4466 (16)	0.048 (6)*	
H1N3	−0.064 (6)	−0.199 (2)	0.4587 (17)	0.066 (8)*	
H1N1	0.481 (6)	0.231 (2)	0.5712 (16)	0.060 (7)*	

### Atomic displacement parameters (Å^2^)

**Table:**

	*U*^11^	*U*^22^	*U*^33^	*U*^12^	*U*^13^	*U*^23^
O1	0.0552 (9)	0.0305 (7)	0.0471 (7)	0.0065 (6)	−0.0067 (7)	0.0062 (6)
N2	0.0372 (8)	0.0285 (8)	0.0379 (8)	−0.0002 (7)	0.0034 (7)	0.0003 (6)
N3	0.0488 (10)	0.0313 (9)	0.0485 (9)	−0.0036 (8)	−0.0060 (9)	0.0006 (8)
C8	0.0338 (10)	0.0300 (9)	0.0356 (9)	0.0003 (9)	0.0061 (8)	0.0005 (7)
C6	0.0382 (10)	0.0273 (9)	0.0374 (9)	0.0020 (8)	0.0044 (9)	0.0014 (7)
C7	0.0342 (9)	0.0237 (8)	0.0371 (8)	0.0007 (8)	0.0047 (8)	0.0004 (7)
N1	0.0488 (10)	0.0234 (8)	0.0494 (9)	−0.0043 (7)	0.0025 (9)	0.0039 (7)
C1	0.0448 (11)	0.0346 (10)	0.0430 (10)	0.0047 (10)	0.0000 (10)	0.0022 (8)
C4	0.0437 (12)	0.0425 (11)	0.0517 (11)	−0.0096 (11)	0.0048 (10)	−0.0088 (9)
C2	0.0454 (12)	0.0545 (12)	0.0407 (10)	0.0126 (11)	−0.0036 (10)	−0.0016 (9)
C5	0.0365 (11)	0.0313 (9)	0.0393 (9)	−0.0031 (8)	0.0062 (8)	−0.0005 (7)
C3	0.0402 (12)	0.0627 (14)	0.0443 (10)	0.0001 (11)	−0.0030 (10)	−0.0113 (10)

### Geometric parameters (Å, °)

**Table:**

O1—C6	1.236 (2)	N1—C5	1.404 (3)
N2—C7	1.299 (2)	N1—H1N1	0.90 (2)
N2—N3	1.326 (2)	C1—C2	1.384 (3)
N3—H2N3	0.88 (2)	C1—H1A	0.9300
N3—H1N3	0.91 (3)	C4—C5	1.375 (3)
C8—C1	1.385 (3)	C4—C3	1.378 (3)
C8—C5	1.401 (3)	C4—H4A	0.9300
C8—C7	1.455 (3)	C2—C3	1.388 (3)
C6—N1	1.356 (3)	C2—H2B	0.9300
C6—C7	1.487 (2)	C3—H3A	0.9300
			
C7—N2—N3	119.15 (15)	C2—C1—C8	119.33 (19)
N2—N3—H2N3	118.5 (15)	C2—C1—H1A	120.3
N2—N3—H1N3	112.9 (15)	C8—C1—H1A	120.3
H2N3—N3—H1N3	128 (2)	C5—C4—C3	118.1 (2)
C1—C8—C5	119.17 (19)	C5—C4—H4A	120.9
C1—C8—C7	134.24 (18)	C3—C4—H4A	120.9
C5—C8—C7	106.57 (15)	C1—C2—C3	120.3 (2)
O1—C6—N1	126.82 (17)	C1—C2—H2B	119.8
O1—C6—C7	126.73 (17)	C3—C2—H2B	119.8
N1—C6—C7	106.45 (16)	C4—C5—C8	121.84 (18)
N2—C7—C8	126.17 (16)	C4—C5—N1	128.95 (18)
N2—C7—C6	127.29 (17)	C8—C5—N1	109.21 (17)
C8—C7—C6	106.52 (15)	C4—C3—C2	121.2 (2)
C6—N1—C5	111.25 (15)	C4—C3—H3A	119.4
C6—N1—H1N1	123.2 (16)	C2—C3—H3A	119.4
C5—N1—H1N1	125.3 (17)		
			
N3—N2—C7—C8	−179.67 (18)	C7—C8—C1—C2	−178.4 (2)
N3—N2—C7—C6	−0.8 (3)	C8—C1—C2—C3	−0.4 (3)
C1—C8—C7—N2	−3.2 (4)	C3—C4—C5—C8	−0.2 (3)
C5—C8—C7—N2	178.52 (18)	C3—C4—C5—N1	178.8 (2)
C1—C8—C7—C6	177.8 (2)	C1—C8—C5—C4	0.6 (3)
C5—C8—C7—C6	−0.5 (2)	C7—C8—C5—C4	179.15 (17)
O1—C6—C7—N2	1.1 (3)	C1—C8—C5—N1	−178.57 (17)
N1—C6—C7—N2	−178.18 (18)	C7—C8—C5—N1	0.0 (2)
O1—C6—C7—C8	−179.83 (17)	C6—N1—C5—C4	−178.5 (2)
N1—C6—C7—C8	0.8 (2)	C6—N1—C5—C8	0.6 (2)
O1—C6—N1—C5	179.82 (17)	C5—C4—C3—C2	−0.5 (3)
C7—C6—N1—C5	−0.9 (2)	C1—C2—C3—C4	0.8 (3)
C5—C8—C1—C2	−0.2 (3)		

### Hydrogen-bond geometry (Å, °)

**Table:**

*D*—H···*A*	*D*—H	H···*A*	*D*···*A*	*D*—H···*A*
N3—H2N3···O1	0.88 (2)	2.09 (2)	2.784 (2)	135 (2)
N3—H1N3···N2^i^	0.91 (2)	2.20 (3)	3.098 (2)	169 (2)
N1—H1N1···O1^ii^	0.90 (2)	1.98 (2)	2.866 (2)	168 (3)

Symmetry codes: (i) *x*−1/2, −*y*−1/2, −*z*+1; (ii) *x*+1/2, −*y*+1/2, −*z*+1.
